# Hypoglycemic effects of tramadol analgesia in hospitalized patients: a case-control study

**DOI:** 10.1186/s40200-017-0311-9

**Published:** 2017-07-24

**Authors:** Larry K. Golightly, Bonita A. Simendinger, Gerard R. Barber, Nancy M. Stolpman, Steven D. Kick, Michael T. McDermott

**Affiliations:** 1 University of Colorado Hospital, Aurora, CO USA; 2 Division of Clinical Pharmacy, University of Colorado Skaggs School of Pharmacy and Pharmaceutical Sciences, Aurora, CO USA; 3 Health Sciences Library/Center for Drug Information, Education, and Evaluation, University of Colorado Hospital, Anschutz Medical Campus Box A-003, 12950 East Montview Boulevard, Aurora, CO 80045-2515 USA; 4 Division of General Internal Medicine, University of Colorado School of Medicine, Aurora, CO USA; 5 Division of Endocrinology Diabetes and Metabolism, University of Colorado School of Medicine, Aurora, CO USA

**Keywords:** Diabetes, Neuropathy, Tramadol, Hypoglycemia, Oxycodone

## Abstract

**Background:**

In outpatient populations, hypoglycemia has been associated with tramadol. We sought to determine the magnitude of risk for hypoglycemia associated with tramadol use in hospitalized patients.

**Methods:**

During a 2-year period of observation, adult inpatients who received ≥1 dose of tramadol were identified and their medical records were reviewed. Patients were included if they had blood or plasma glucose (BG) concentrations measured on at least two occasions within five days after the initial administration of tramadol. A contemporary comparator group of hospitalized oxycodone recipients was similarly reviewed.

**Results:**

Tramadol was administered to 2927 patients who met inclusion criteria. Among these, hypoglycemia (BG ≤70 mg/dL) was documented in 22 (46.8%) of 47 patients with type 1 diabetes, 113 (16.8%) of 673 patients with type 2 diabetes, and 103 (4.7%) of 2207 patients who did not have a diabetes mellitus diagnosis. In those without a diabetes diagnosis, the causality association between hypoglycemia and tramadol use was probable in 77 patients (3.5%). By comparison, hypoglycemia was documented in 8 (1.1%) of 716 matched oxycodone recipients without diabetes (*p* = 0.002). As compared with tramadol recipients who did not develop low BG concentrations, those who experienced tramadol-related hypoglycemia were relatively young (mean age 52.0 versus 59.8 years; *p* = 0.027) and predominantly female (74.0% versus 59.8%; *p* = 0.012).

**Conclusions:**

Tramadol use was causally associated with hypoglycemia in hospitalized patients. The proportion of patients without diabetes who developed hypoglycemia was higher among those who received tramadol than among those who received oxycodone.

**Trial registration:**

Colorado Multiple Institutional Review Board Protocol № 15–2215. Registered/approved 8 December 2015.

## Background

Tramadol is a centrally-acting analgesic medication that has been in clinical use for several decades. The antinociceptive effects of tramadol are imparted by drug and metabolite binding to μ-opioid receptors and inhibition of neuronal reuptake of serotonin and norepinephrine [1]. In pharmacological models, both of these actions have been shown to not only enhance insulin effects but also directly promote glucose utilization [2, 3]. Clinically, this may result in decreased blood glucose concentrations. Individual case reports have highlighted severe hypoglycemia as a possible consequence of tramadol overdose [4] and recent epidemiological surveys documented increased risk for symptomatic hypoglycemia among diabetic and nondiabetic outpatients taking therapeutic doses of tramadol [5, 6]. However, the overall magnitude of hypoglycemic risk associated with tramadol remains unclear and no recommendations for patient monitoring are currently available.

The aims of this investigation were to determine (1) if hospitalized patients are affected by tramadol-related hypoglycemic effects; (2) whether tramadol administration imparts increased risk for hypoglycemia as compared with use of other opioid analgesic medications; and (3) which patient characteristics or comorbidities confer increased risk for tramadol-related hypoglycemia.

## Methods

**Fig. 1:**
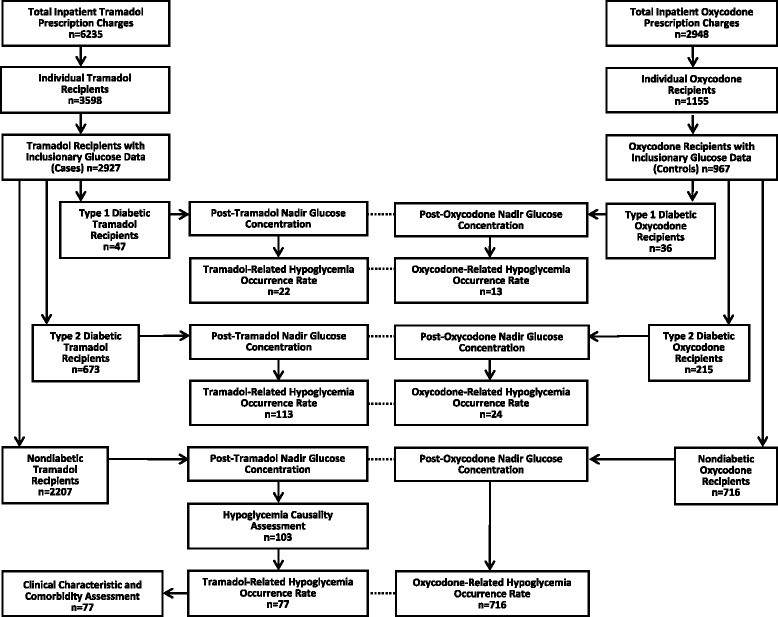
Study design

Our study design is diagrammed in Fig. 1. Briefly, we performed a retrospective case-control observational study.

### Data source and sample selection

This study was conducted at the University of Colorado Hospital. This is a 533-bed academic medical center that serves as the primary teaching hospital in the Rocky Mountain region of the United States. The study was conducted with approval of our local ethics committee and the hospital’s Research Support Service.

Hospitalized patients greater than 18 years of age who received ≥1 dose of oral tramadol for management of acute or chronic pain during the period of observation from July 1, 2013 to June 30, 2015 were identified through a search of computerized inpatient prescription data (Epic Willow, Epic Systems Corporation, Verona Wisconsin USA). Individual medical records of these tramadol recipients were retrospectively examined. Patients were included if they had ≥2 laboratory plasma glucose or point-of-care blood glucose measurements (hereafter collectively represented as BG) performed within 5 days after the first recorded in-hospital administration of tramadol. Demographic information was recorded, including the presence or absence of documentation of a diabetes mellitus diagnosis in the patient’s history and/or current problem list, as well as relevant BG data. The BG measurement of interest was the lowest BG concentration recorded during the first 5 days after the initial in-hospital tramadol administration.

A comparator group of patients was established. Patients in the comparator group were similarly identified by a review of inpatient prescription data. Adult patients were included in the comparator group if they received ≥1 dose of oral immediate-release oxycodone while hospitalized from December 1 through December 31, 2015. Patients were included in the comparator group if they had ≥2 BG measurements determined within 5 days after the first recorded in-hospital administration of oxycodone. Demographic information was recorded, including the presence or absence of documentation of a diabetes mellitus diagnosis in the patient’s history and/or current problem list. Relevant BG data were recorded. The measurement of interest was the lowest BG concentration recorded during the first 5 days after the initial in-hospital oxycodone administration.

Patients who received either tramadol or oxycodone comparator therapy were aggregated according to diabetes mellitus classification as either type 1 (T1DM) or type 2 (T2DM). Patients who did not have documentation of a diabetes mellitus diagnosis were respectively aggregated to tramadol and oxycodone comparator groups without diabetes.

### Factors influencing blood glucose concentrations

All medical records of included patients without a diabetes mellitus diagnosis were examined to identify factors that could contribute to development of hypoglycemia. These factors included not only clinical and nutritional conditions but also acute interventions with known potential to alter glycemic control and depress BG concentrations. Tramadol recipients without a diabetes mellitus diagnosis and low BG concentrations were further evaluated with a standardized algorithmic tool to determine causal relationships between tramadol administration and hypoglycemia [7]. In general, documentation of the presence of any factor capable of altering glycemic control degraded causality of hypoglycemia temporally associated with tramadol administration from probable to merely possible. Tramadol recipients without diabetes whose medical records displayed any such factor were excluded from intragroup comparisons of the occurrence rate for hypoglycemia (expressed as the percentage of patients experiencing hypoglycemia occurring during the 5-day period following the first in-hospital tramadol administration). In similar fashion, oxycodone recipients without diabetes with low BG concentrations were excluded from occurrence rate comparisons for hypoglycemia if their medical records contained documentation of the presence of any factor that may have contributed to the development of hypoglycemia.

Clinical factors that could potentially contribute to the occurrence of hypoglycemia were evaluated. Among patients without a diabetes mellitus diagnosis whose causality relationship between tramadol administration and hypoglycemia was graded as probable, individual patient characteristics, primary and secondary diagnoses, and estimated severity of various comorbidities [8] were analyzed for comparative prevalence and strengths of association.

### Outcomes

The primary outcome event was objective documentation of hypoglycemia occurring within 5 days after the initiation of tramadol or oxycodone therapy. Consistent with alerting values in current guidelines [9, 10], hypoglycemia was defined as having at least one recorded BG concentration ≤ 70 mg/dL. Percentage occurrence rates for hypoglycemia were compared between patients with T1DM and T2DM and patients without a diabetes mellitus diagnosis who received either tramadol or oxycodone.

Additional outcomes related to hypoglycemia were investigated. To determine whether the occurrence rate for hypoglycemia after tramadol administration was different than the occurrence rate for hypoglycemia that occurs in the general hospital population, the post-tramadol percentage occurrence rates for hypoglycemia in patients with and without diabetes occurring during the 2-year period of observation were compared with the overall percentage occurrence rate for hypoglycemia documented in a convenience sample that contained hospital-wide results of all point-of-care BG concentrations measured from October 1 through November 30, 2015. BG measurements in the convenience sample were sourced from patients with and without a diabetes mellitus diagnosis.

### Statistical analysis

Continuous data are expressed as the mean ± standard deviation (SD). Proportions and ratios are reported as percentages, multiples, or fractions with 95% confidence Intervals (CI). Nominal occurrence rates for hypoglycemia were compared by construction of 2 × 2 contingency tables and statistical testing with Chi-squared. Continuous variables were tested as discrete populations with Student’s *t*.

Relative risk ratio (RRR) was calculated as the ratio of the probability of hypoglycemia occurring in the tramadol-exposed group without diabetes to the probability of hypoglycemia occurring in the oxycodone-exposed comparator group without diabetes. Number needed to harm (NNH) was calculated as the inverse of the probability of hypoglycemia occurring in the tramadol group without a diabetes mellitus diagnosis minus the probability of hypoglycemia occurring in the oxycodone comparator group without diabetes.

Statistical analyses were conducted with SAS version 9.2 (SAS Institute, Cary, North Carolina USA). Strengths of associations were assessed with hierarchical clustering analysis [11].

## Results

During the 2-year period of observation, a total of 3588 patients received at least one dose of oral tramadol. Among these, 2927 patients (81.6%) had inclusionary BG data available. As shown in Table 1, 47 patients (1.6%) had T1DM, 673 patients (23.0%) had T2DM, and 2207 patients (75.4%) did not have a diabetes mellitus diagnosis. Compared with patients without diabetes (whose mean age was 57.1 ± 18.5 years), patients with T1DM were younger (mean age 43.6 ± 16.8 years; *p* < 0.001) and those with T2DM were older (mean age 62.3 ± 13.7 years; *p* < 0.001).

**Table 1:** Clinical characteristics and post-treatment BG concentrations in tramadol and oxycodone recipients

	Type 1 Diabetes		Type 2 Diabetes		No Diabetes Diagnosis	
	Tramadol	Oxycodone	Tramadol	Oxycodone	Tramadol	Oxycodone
	*n* = 47	*n* = 36	*n* = 673	*n* = 215	*n* = 2207	*n* = 716
Age, yr	43.6 ± 16.8	47.7 ± 14.0	62.3 ± 13.7	59.2 ± 13.8	57.1 ± 18.5^†^	51.6 ± 16.9
Male gender, n (%)	23 (48.9)	17 (47.2)	287 (42.6)	97 (45.1)	882 (40.0)^†^	369 (51.5)
Mean Nadir BG, mg/dL	81.7 ± 36.5	88.9 ± 48.7	99.8 ± 31.2	100.7 ± 27.8	91.9 ± 16.4^†^	98.1 ± 19.9
BG ≤70 mg/dL, n (%)	22 (46.8)	13 (36.1)	113 (16.8)	24 (11.2)	77 (3.5)*	8 (1.1)

BG indicates blood glucose. Plus-minus values are mean ± SD. *p < 0.01 vs oxycodone. ^†^ *p* < 0.001 vs oxycodone

Among 967 comparator oxycodone recipients, 36 (3.7%) had T1DM, 215 (22.2%) had T2DM, and 716 (74.0%) did not have a diabetes mellitus diagnosis. With the exceptions that oxycodone recipients without a diabetes mellitus diagnosis were younger (51.6 ± 16.9 years versus 57.1 ± 18.5 years; *p* < 0.001) and a greater proportion were male (51.5% versus 40.0%; *p* < 0.001), comparator patients were generally well matched to those in the tramadol group.

### Diagnosis and treatment effects on blood glucose concentrations

As compared with tramadol recipients without a diabetes mellitus diagnosis (whose lowest mean BG concentration measured within 5 days after the first in-hospital tramadol administration was 91.9 ± 16.4 mg/dL), mean nadir post-tramadol BG concentrations were lower in patients with T1DM (81.7 ± 36.5 mg/dL; *p* < 0.001). As compared with patients without diabetes, mean nadir post-tramadol BG concentrations were higher in patients with T2DM (99.8 ± 31.2 mg/dL; *p* < 0.001).

Mean nadir post-oxycodone BG concentrations were similar in comparator oxycodone recipients without diabetes (98.1 ± 19.9 mg/dL) and oxycodone recipients with T2DM (100.7 ± 27.8 mg/dL; *p* = 0.122). As compared with patients without diabetes, mean nadir post-oxycodone BG concentrations were lower in patients with T1DM (88.9 ± 48.7 mg/dL; *p* < 0.001). Similarly, mean nadir post-oxycodone BG concentrations were lower in patients with T1DM than in those with T2DM (*p* < 0.001). Thusly, from these determinations no clear pattern of association between a diabetes mellitus diagnosis and BG concentrations was discernable for either tramadol or oxycodone.

The lowest mean BG concentration measured within 5 days after the first in-hospital medication administration was compared between the tramadol and oxycodone groups. No appreciable difference in mean nadir BG concentrations was identified between patients with T1DM who received tramadol (81.7 ± 36.5 mg/dL) or oxycodone (88.9 ± 48.7 mg/dL; *p* = 0.381). Similarly, no difference in mean nadir BG concentrations was identified between patients with T2DM who received tramadol (99.8 ± 31.2 mg/dL) or oxycodone (100.7 ± 27.8 mg/dL; *p* = 0.122). However, in patients without a diabetes diagnosis, post-treatment mean nadir BG concentrations were lower in those who received tramadol (91.9 ± 16.4 mg/dL) than in those who received oxycodone (98.1 ± 19.9 mg/dL; *p* < 0.001).

### Treatment effects on hypoglycemia

Hypoglycemia, the primary outcome event, was reported after tramadol administration in patients with and without diabetes. However, largely because of the frequent use of antidiabetic treatments and medications, the percentage occurrence of post-tramadol hypoglycemia in patients with either T1DM (46.8%) or T2DM (16.8%) was higher than the occurrence of post-tramadol hypoglycemia in patients without a diabetes mellitus diagnosis (4.7%; *p* < 0.001 for both comparisons). The occurrence rate for post-tramadol hypoglycemia was also higher in patients with T1DM than in patients with T2DM (*p* < 0.001).

Assessments of causal relationships between tramadol use and hypoglycemia were performed in all patients without a diabetes mellitus diagnosis in whom post-treatment hypoglycemia was documented. Among 103 tramadol recipients without a diabetes mellitus diagnosis who experienced hypoglycemia, an alternative potentially causal factor for hypoglycemia was present in 26 patients. These factors included postoperative or total parenteral nutrition (TPN)-related prandial and/or correction subcutaneous insulin lispro coverage for hyperglycemia (*n* = 10), concomitant β-adrenergic blocker therapy (*n* = 10), concomitant insulin and β-adrenergic blocker therapy (*n* = 2), ongoing patient evaluation for chronic post-bariatric surgical hypoglycemia (*n* = 2), *nil per os* (NPO) nutritional status (*n* = 1), and acute treatment of hyperkalemia with intravenous regular insulin and 50% dextrose (*n* = 1). An alternative causal factor for hypoglycemia was not present in the remaining 77 patients in this group. Therefore, with regard to the entire cohort of patients without diabetes who received tramadol, a causal relationship between hypoglycemia and tramadol administration was possible in 1.2% of patients (26/2207) and probable in 3.5% (77/2207).

**Fig. 2:**
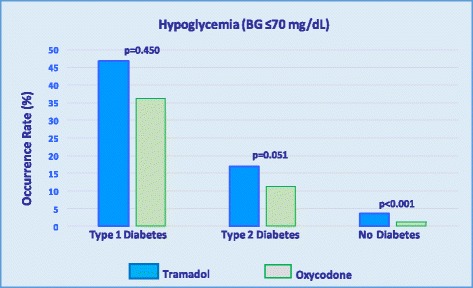
Occurrence rates for hypoglycemia in tramadol and oxycodone recipients

Percentage occurrence rates of hypoglycemia were compared among inpatients who received either tramadol or oxycodone. As illustrated in Fig. 2, among patients with T1DM, post-treatment hypoglycemia was documented in 46.8% of those who received tramadol and in 36.1% of those who received oxycodone (*p* = 0.450). In patients with T2DM, post-treatment hypoglycemia occurred in 16.8% of tramadol recipients and in 11.2% of oxycodone recipients (*p* = 0.051). Although hypoglycemia attributable to tramadol occurred in 3.5% of patients without diabetes, hypoglycemia associated with oxycodone administration occurred in 1.1% of patients without diabetes (*p* < 0.001).

Using oxycodone effects as a reference, the occurrence rate for hypoglycemia in patients without diabetes corresponds to a tramadol RRR of 3.12 (95% CI 1.53 to 6.51). This corresponds to an NNH of 42.2 (95% CI 28.9 to 77.8). Thus, for every 42 inpatients without diabetes who are exposed to tramadol, one will develop hypoglycemia who otherwise would not have been harmed.

**Fig. 3:**
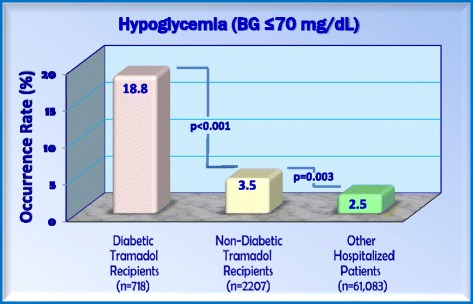
Occurrence rates for hypoglycemia in tramadol recipients and other hospitalized patients

To determine whether the occurrence of hypoglycemia occurring after tramadol administration was different than the occurrence of hypoglycemia occurring in the general hospital population, the post-tramadol percentage occurrence rates for hypoglycemia were compared to the occurrence reported in a contemporary hospital-wide survey of 61,083 point-of-care BG measurements. As illustrated in Fig. 3, the occurrence rate for hypoglycemia in the combined groups of tramadol recipients with T1DM and T2DM (18.8%) was higher than the rate reported in tramadol recipients without diabetes (3.5%; *p* < 0.001). Likewise, the occurrence rate for hypoglycemia in both of these groups who received tramadol was higher than the occurrence rate of hypoglycemia in the general hospital population (2.5%; *p* < 0.001 and *p* = 0.003, respectively).

### Patient characteristics associated with increased risk for hypoglycemia

Patients without a diabetes mellitus diagnosis who developed hypoglycemia probably caused by tramadol were reviewed with regard to common and distinguishing clinical characteristics. As compared with tramadol recipients without diabetes who did not experience hypoglycemia (*n* = 2130), hypoglycemic patients (*n* = 77) were relatively young (mean age 52.0 ± 17.19 versus 57.3 ± 18.61 years; *p* = 0.027). As further compared to patients without hypoglycemia, hypoglycemic patients were predominantly female (74.0% versus 59.8%; *p* = 0.012).

The associations between tramadol-related hypoglycemia and age and gender in patients without a diabetes diagnosis were identified with hierarchical clustering analysis [11]. However, hierarchical clustering analyses failed to disclose associations between hypoglycemia in tramadol recipients without diabetes and other distinguishing patient characteristics including primary diagnosis, acuity level, tramadol dose, concurrent use of other opioids, concurrent use of antidepressants, source of pain, recent surgery or trauma, and presence or severity of comorbidities including kidney, liver, immunological, hematological, cardiovascular, respiratory, and malignant disorders.

## Discussion

Our investigation revealed that tramadol use exerted a negative influence on glucose levels in hospitalized patients with a resultant increase in occurrence rates and overall risk for objectively defined hypoglycemia. As compared with use of other opioids in patients without a diabetes mellitus diagnosis, tramadol exposure was associated with a significant increase in the percentage occurrence rate for hypoglycemia. The hypoglycemic effects of tramadol appeared to be most prominent among female patients of relatively young age. These findings are generally congruent with limited information currently available regarding this adverse drug effect.

First introduced into clinical medicine in the early 1990s, tramadol is an atypical opioid analgesic medication. Due to prominent activity against neuropathic pain [12], tramadol is generally regarded as a third-tier agent for pain management in patients with diabetic neuropathy [13, 14]. In the United States, tramadol currently ranks among the top 25 most frequently prescribed drugs and its popularity appears to be increasing [15]. This is believed to be the result of overall trends in opioid use and misuse (the so-called opioid epidemic) [16, 17] and because of widely held perceptions that tramadol is a relatively inexpensive “non-narcotic” pain reliever with minimal potential for tolerance, dependence, or abuse [18, 19]. However, reflective of its prominent morphine-like actions and proven potential for possible diversion and misuse or abuse [20, 21], tramadol (a previously unclassified agent) was recently reclassified as a Schedule IV controlled substance with limited availability by prescription in the United States.

The antinociceptive effects of tramadol are primarily enacted through two complementary actions. First, early pharmacological studies showed that tramadol and its primary metabolite (*O*-desmethyltramadol, M1) nonselectively bind to μ-, κ-, and δ-opioid receptors [22]. Although the receptor binding affinity of tramadol for μ-opioid receptors is relatively weak, M1 shows direct μ-opioid receptor affinity that is 300 times greater than its parent [23, 24]. Thus, μ-opioid analgesic activity is largely derived from M1, with tramadol essentially serving as a prodrug. Secondly, animal and human volunteer studies have demonstrated that tramadol and M1 inhibit neuronal reuptake of monoamine neurotransmitters. Reuptake of serotonin (5-hydroxytryptamine) is inhibited in postsynaptic neurons as is reuptake of norepinephrine released from presynaptic α_2_-adrenoceptors [25–27]. These serotonergic and noradrenergic effects contribute to the analgesic effect of tramadol by inhibiting transmission of pain signals within the central nervous system.

Laboratory investigations have shown that the receptor interactions described above can negatively affect glucose concentrations. In diabetic rats, tramadol exposure produced a dose-dependent lowering of plasma glucose that was induced by a non-insulin mediated increase in peripheral glucose utilization and a decrease in hepatic gluconeogenesis [28]. In a pancreatectomized rodent model, tramadol administration elicited a hypoglycemic response derived from enhanced hepatic glucose utilization that was mediated by increased insulin signaling in the cerebral cortex and hypothalamus [3]. In both of these animal studies, the hypoglycemic effects of tramadol were reversed by the narcotic antagonist naloxone, suggesting that these actions were related to blockade of μ-opioid receptors. Additional evidence has shown that μ-opioid receptor activation is associated with elevation of circulating levels of β-endorphin that ameliorates the post-receptor insulin signaling cascade that increases insulin resistance [2].

In similar fashion, administration of serotonin in diabetic rats was associated with hypoglycemic effects that were mediated by an increase in β-endorphin levels and a resultant increase in peripheral glucose utilization [29]. Investigations in rodents additionally demonstrated that administration of exogenous serotonin increased insulin levels and blunted glucagon secretion in response to hypoglycemia [30, 31]. Correspondingly, it has been postulated that inhibition of neuronal serotonin reuptake by tramadol may be at least partially responsible for tramadol-related decreases in glucose concentrations. Thus, plausible pharmacological mechanisms exist for clinically relevant hypoglycemic effects that may result from tramadol administration.

The first case report of tramadol-related hypoglycemia was published in 2006 [32]. This report from the French Center for Pharmacovigilance described two patients—an 88 year-old nondiabetic female with normal renal function and an 8 year-old girl with diabetes—who developed symptoms requiring treatment for BG values of 38 mg/dL and 51 mg/dL, respectively, after receiving single doses of tramadol. Subsequently, individual case reports similarly described five additional patients (two of whom had diabetes) that suffered severe symptomatic hypoglycemia following tramadol administration [33–35]. Additional reports have described the occurrence of severe hypoglycemia in subjects without diabetes following tramadol overdose [4, 36].

Epidemiological community surveys from France and the United Kingdom have highlighted risk for hypoglycemia in outpatients receiving tramadol. A report from the French Association of Regional Pharmacovigilance Centers identified 43 cases of hypoglycemia associated with tramadol that occurred during the years from 1997 to 2010. Among affected patients, the median age was 69 years, at least one risk factor for hypoglycemia was present in most patients (including a diabetes mellitus diagnosis in 42%), the onset of hypoglycemia occurred after a median of 5 days’ use, and the reported mean BG concentration was 45 mg/dL [37]. A follow-up report [5] from this pharmacovigilance system described an additional 8 cases of hypoglycemia associated with tramadol in the 12-month period following publication of the initial report.

Most recently, a 14-year nested case-control analysis [6] of 334,034 patients who received a prescription for oral codeine or tramadol for noncancer pain revealed that hospitalization for hypoglycemia occurred within 30 days in 1105 patients. Of these, 112 (10.1%) were fatal. The overall event rates for hospitalization for hypoglycemia were 3.0 (95% CI 1.3 to 6.0) and 0.7 (95% CI 0.4 to 1.1) per 10,000 person-months in tramadol and codeine users, respectively. As compared with codeine, tramadol use was associated with a greater than 3-fold increase in risk for hospitalization as well as an increased risk for fatal hypoglycemia. Remarkably, the likelihood of developing hypoglycemia requiring hospitalization was higher in nonusers than in users of antidiabetic medications.

Oxycodone, the comparator medication in this investigation, is a relatively selective μ-opioid receptor-specific ligand with pure agonist properties [38]. This selectivity is believed to confer differing effects on glycemic control as compared with other opioids such as methadone and tramadol. In pharmacological models, oxycodone has been shown to be devoid of glucose lowering activity [39]. This is consistent with our findings that suggest oxycodone exerted little or no effect on BG concentrations.

Our study has limitations. This investigation was performed in a single institution using an uncontrolled design. No institutional mandate was in place with regard to requirements for adequacy or completeness of individual monitoring of glycemic control. Diagnostic categorization depended on information available within the snapshot history and problem list for each patient, with assumptions that these sources are comprehensive and up to date. The relationships between tramadol or oxycodone administration and hypoglycemia were assessed with objective measures only. No subjective accounting was included for the presence, absence, or severity of symptoms associated with hypoglycemia and the need for treatment intervention for hypoglycemic episodes was not evaluated. Individual differences among patients’ antidiabetic treatment regimens and their corresponding associations with hypoglycemic tendencies were not comparatively assessed. Although the experience with tramadol spanned a 2-year period, our investigation suffers from sample sizes that are relatively small. Given the percentage occurrence rates for hypoglycemia documented among tramadol and oxycodone recipients with T2DM (16.8% and 11.2%, respectively), power calculations reveal that attainment of significant differences between these rates would require evaluation of patient populations that are 35% larger. Among tramadol and oxycodone recipients with T1DM (whose respective occurrence rates for hypoglycemia were 46.8% and 36.1%), attainment of significant differences in rates of occurrence of hypoglycemia would require evaluation of patient populations that are nearly 8-fold larger in size. Nonetheless and despite these limitations, this study examined an aspect of contemporary acute care that is representative of patient management methods used in a broad array of modern hospitals.

## Conclusion

Tramadol use was associated with hypoglycemia in hospitalized patients. In our population of tramadol recipients without a diabetes mellitus diagnosis, the occurrence rate for hypoglycemia was approximately 4%. Patients with diabetes appear to be at heightened risk for tramadol-related hypoglycemia. Accordingly, BG monitoring should be performed in patients with diabetes. BG monitoring also should be strongly considered for hospitalized patients who do not have diabetes when tramadol therapy is initiated.

## Abbreviations

BG: Blood glucose
CI: Confidence interval
M1: *O*-Desmethyltramadol
NNH: Number needed to harm
NPO: *nil per os* (nothing by mouth)
OR: Odds ratio
RRR: Relative risk ratio
SD: Standard deviation
T1DM: Type 1 diabetes mellitus
T2DM: Type 2 diabetes mellitus
TPN: Total parenteral nutrition
